# Prefrontal correlates of fear generalization during endocannabinoid depletion

**DOI:** 10.1172/JCI179881

**Published:** 2025-03-27

**Authors:** Luis E. Rosas-Vidal, Saptarnab Naskar, Leah M. Mayo, Irene Perini, Rameen Masroor, Megan Altemus, Liorimar Ramos-Medina, S. Danyal Zaidi, Hilda Engelbrektsson, Puja Jagasia, Markus Heilig, Sachin Patel

**Affiliations:** 1 Stephen M. Stahl Center for Psychiatric Neuroscience, Department of Psychiatry and Behavioral Sciences, Northwestern University, Feinberg School of Medicine, Chicago, Illinois, USA.; 2 Hotchkiss Brain Institute and Mathison Centre for Mental Health Research and Education, Department of Psychiatry, Cumming School of Medicine, University of Calgary, Calgary, Alberta, Canada.; 3 Linköping University, Center for Social and Affective Neuroscience, Department of Biomedical and Clinical Sciences, Linköping, Sweden.; 4 Vanderbilt University Medical Center, Department of Psychiatry and Behavioral Sciences, Nashville, Tennessee, USA.

**Keywords:** Clinical Research, Neuroscience, Behavior, Neuroimaging, Psychiatric diseases

## Abstract

Maladaptive fear generalization is one of the hallmarks of trauma-related disorders. The endocannabinoid 2-arachidonoylglycerol (2-AG) is crucial for modulating anxiety, fear, and stress adaptation, but its role in balancing fear discrimination versus generalization is not known. To address this, we used a combination of plasma endocannabinoid measurement and neuroimaging in a childhood maltreatment–exposed and –nonexposed mixed population, combined with human and rodent fear-conditioning models. Here we show that 2-AG levels were inversely associated with fear generalization at the behavioral level in both mice and humans. In mice, 2-AG depletion increased the proportion of neurons that respond to, and the similarity of neuronal representations for, both threat-predictive and neutral stimuli within prelimbic prefrontal cortex neuronal ensembles. In humans, increased dorsolateral prefrontal cortical–amygdala resting-state connectivity was inversely correlated with fear generalization. These data provide convergent cross-species evidence that 2-AG is a key regulator of fear generalization and further support the notion that 2-AG deficiency could represent a trauma-related disorder-susceptibility endophenotype.

## Abstract 2

The authors show in both mice and humans that low levels of the endocannabinoid 2-AG is associated with increased generalization of fear at the behavioral and neuronal representation levels.

## Introduction

Generalization of conditioned associations is advantageous for survival, as it maximizes goal attainment while minimizing harm, based on rewarding and dangerous experiences, respectively. However, overgeneralization is a hallmark and often debilitating symptom of psychiatric disorders, including posttraumatic stress disorder (PTSD) (1–5), and underlies the broadening of sensory stimuli capable of inducing hyperarousal and avoidance symptoms central to the disorder. The amygdala and the medial prefrontal cortex (mPFC) are crucial for balancing fear memory generalization and discrimination (3, 6–8). Inactivation or lesions of the mPFC increase contextual fear generalization (9, 10), and generalization states correlate with loss of mPFC-amygdala entrainment (11). Specifically, in rodents, the prelimbic prefrontal cortex (PL) plays a crucial role in fear expression (12, 13) and opposes generalization of the conditioned stimuli to the neutral stimuli (8, 14, 15). It is not known, however, how generalization between discrete fear-associated and neutral stimuli is represented by neuronal ensembles within the PL.

The endocannabinoid system has been implicated in the regulation of stress, anxiety, and fear states and has been proposed as a promising target for stress-related psychiatric disorders (16). One of the endocannabinoids, 2-arachidonoylglycerol (2-AG), is synthesized primarily by diacylglycerol-lipase α (DAGLα) in the brain, and inhibition of this enzyme in experimental animals is associated with increased anxiety, decreased resiliency to stress, and impaired fear extinction (17–20). While 2-AG has been implicated in stress resiliency and found to be lower in the plasma of subjects with PTSD (ref. 21; but see ref. 22), the association between 2-AG levels and fear generalization has not to our knowledge been systematically investigated in humans or animal models.

In this study, we use fear conditioning in both mice and humans in combination with plasma 2-AG measurements, pharmacological manipulations, human brain imaging, and single-neuron calcium imaging to establish the role of 2-AG levels in the regulation of fear generalization and 2-AG’s effect on prefrontal neural correlates. We show that 2-AG levels predicted and regulated fear memory generalization and that specificity to sensory stimuli within PL neuronal ensembles deteriorated in 2-AG–deficient states. Furthermore, resting-state prefrontal-amygdala connectivity was inversely correlated with fear generalization. These data provide insights into 2-AG regulation of fear generalization in humans and mice and reveal how 2-AG–deficient states affect cellular correlates of fear generalization in PL neurons.

## Results

### Peripheral 2-AG levels are inversely correlated with conditioned fear generalization in humans.

**Figure 1:**
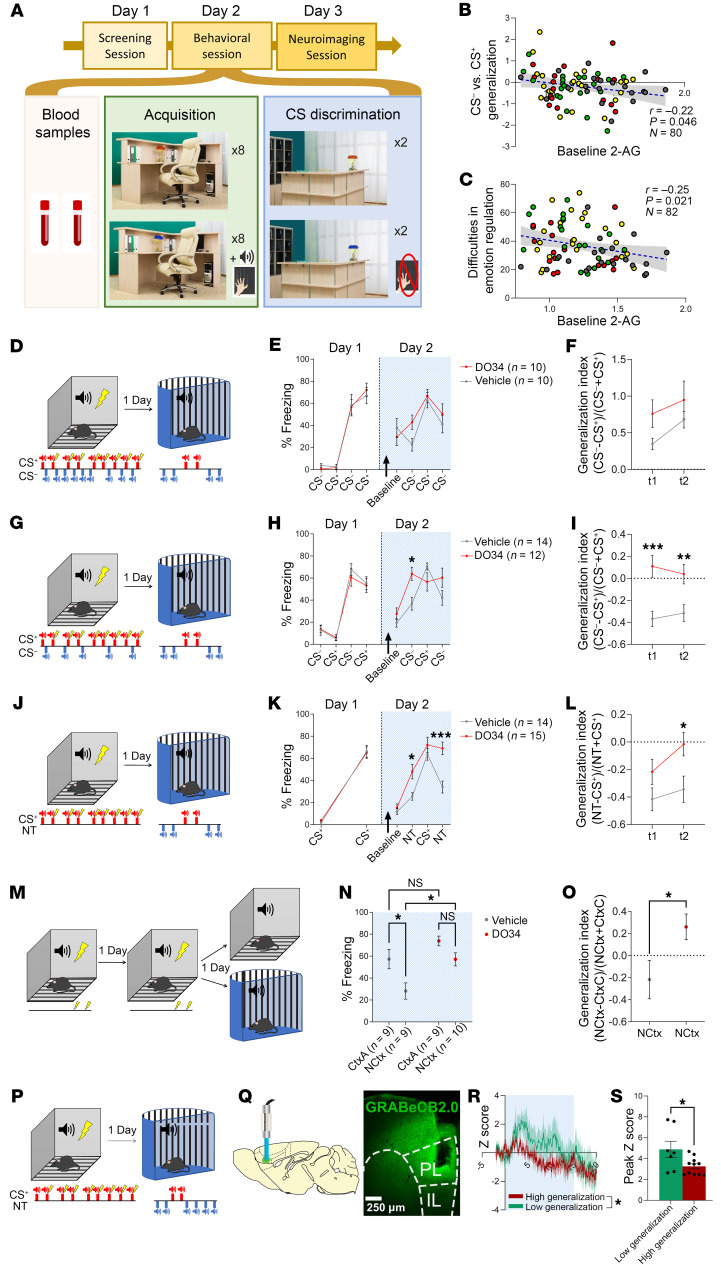
Decreased levels of 2-AG are associated with increased generalization to neutral stimuli and self-reported emotional regulation. (**A**) Experiment schematic. (**B**) Generalization between CS^+^ and CS^–^ and (**C**) difficulties in emotional regulation as a function of baseline peripheral 2-AG levels. Scatter plots: yellow, participants with CM and SUD history; red, participants with CM history; green, participants with SUD history; and gray, participants with no CM or SUD history. (**D**) Experiment schematic depicting stimuli for differential fear-conditioning and recall test sessions. (**E**) Percentage of time freezing in response to CS^+^ and CS^–^. Arrow, i.p. injection of vehicle or DO34 before recall test. (**F**) Generalization index for CS^–^ *t_1_* and *t_2_* for vehicle and DO34 groups. (**G**) Experiment schematic depicting stimuli for partial differential fear-conditioning and recall test sessions. (**H**) Percentage of time freezing to CS^+^ and CS^–^. (**I**) Generalization index for CS^–^ *t_1_* and *t_2_* for vehicle and DO34 groups. (**J**) Experiment schematic depicting stimuli for classical fear-conditioning and recall test sessions. (**K**) Percentage of time freezing to CS^+^ and NT. (**L**) Generalization index for NT *t_1_* and *t_2_* for vehicle and DO34 groups. (**M**) Experiment schematic depicting stimuli for contextual fear-conditioning and recall test sessions. (**N**) Percentage of time freezing to conditioning context (CtxA) and novel context (NCtx). (**O**) Generalization index for NCtx relative to freezing to CtxA on conditioning day 2 (CtxC). (**P**) Experiment schematic depicting stimuli for classical fear-conditioning and recall test sessions. (**Q**) Sagittal depiction (left) and coronal section (right) showing optic fiber and GRABeCB2.0 expression in PL. The dashed lines correspond to the subdivisions of the medial prefrontal cortex. (**R**) Average NT response for trials with freezing to tones above 75% (high generalization) and below 25% (low generalization). (**S**) Peak population NT response for high- and low-generalization trials. Repeated-measures or 2-way ANOVA followed by Šídák’s multiple-comparison test or Student’s *t* test where appropriate. *P < 0.05, **P < 0.01, ***P < 0.001.

While preclinical studies clearly implicate 2-AG signaling in the regulation of stress-related behavioral phenotypes, results of clinical studies linking peripheral 2-AG levels to stress-related psychiatric diagnoses are mixed (21–31). To specifically examine the relationship between plasma 2-AG levels and fear learning, expression, and generalization, we combined an experimental model of conditioning in participants together with measures of baseline plasma 2-AG (*n* = 80) and resting-state brain connectivity (*n* = 88). Demographics of study participants are shown in Table 1. Figure 1A depicts the experimental timeline and approach. On day 1, subjects participated in a screening session as described in Methods. On day 2, baseline 2-AG levels were obtained, and subsequently, subjects were exposed to the fear-conditioning protocol. On day 3, a resting-state imaging session was conducted. Using endocannabinoid levels as a covariate in the fear-conditioning analysis, we found a relationship between 2-AG and the ability to distinguish between a conditioned stimulus predictive of an aversive outcome (CS^+^) and CS^–^, associated with the absence of an aversive outcome. Specifically, following acquisition, there was a significant interaction between the startle response to CS^–^ as compared with CS^+^ during fear recall (*F*[1, 78] = 4.10, *P* = 0.046, partial η^2^ = 0.05). Post hoc linear regression with CS^–^ versus CS^+^ generalization [calculated as (CS^–^) – (CS^+^)] as the dependent variable and 2-AG levels as a predictor confirmed that higher 2-AG predicted less generalization to CS^–^ presentations (Pearson’s correlation = 0.223; *P* = 0.046; Figure 1B). Analysis of groups individually confirmed that there was no significant relationship between 2-AG and fear generalization in each group individually (history of childhood maltreatment [CM] and substance use disorders [SUDs], *n* = 24, *P* = 0.08; CM only, *n* = 16, *P* = 0.45; SUDs only, *n* = 20, *P* = 0.12; control, *n* = 20, *P* = 0.88). There was also no difference in the variance of 2-AG levels across groups (Levene’s test of equality of error variance, *P* = 0.53). Similarly, self-reported difficulties in emotion regulation were associated with 2-AG levels, such that lower peripheral levels of 2-AG predicted greater self-reported impairment in emotion regulation (*r* = –0.25, *P* = 0.021, *n* = 82; *F*[1,81] = 5.51, *P* = 0.013, 95% CI –27.0 to –3.44, 1,000 bootstraps; Figure 1C). A subanalysis of startle responses for subjects within the upper and lower thirds of generalization revealed that on average, high generalizers showed lower CS^+^ and higher CS^–^ responses. In contrast, the opposite was observed for low generalizers (stimulus × group interaction: *F*[1, 64] = 105.8, *P* < 0.0001; Supplemental Figure 1; supplemental material available online with this article; https://doi.org/10.1172/JCI179881DS1). We did not observe a significant interaction between anandamide (AEA) and CS^–^ as compared with CS^+^ during fear recall (*F*[1, 84] = 0.28, *P* = 0.60). Importantly, we also did not observe differences between cues during acquisition (cue: *F*[1, 84] = 0.51, *P* = 0.48).

### 2-AG depletion is associated with increased fear generalization in mice.

Our human data indicated an inverse association between peripheral 2-AG levels and fear generalization, suggesting that lowering 2-AG levels could promote fear generalization. To test this hypothesis, we used a variety of fear-conditioning paradigms in mice together with systemic injections of the DAGL inhibitor DO34 to pharmacologically inhibit the production of 2-AG before fear recall (32). Reducing 2-AG levels on day 2 of a differential fear-conditioning paradigm, before fear recall, did not increase freezing in response to CS^–^ or CS^+^ presentation or generalization (Figure 1, D–F, and Supplemental Figure 2, A–C). We hypothesized that our differential fear-conditioning protocol in mice overtrained and minimized the ambiguity of the CS^–^ or became associated as a safety cue, which in turn minimized the effects of reducing 2-AG on fear generalization in mice. To test this hypothesis, we replicated our previous experiment using a partial differential conditioning protocol, whereby mice were presented with 5 CS^–^ tones rather than 9 (Figure 1G and Supplemental Figure 2D). DO34 injections before fear recall increased freezing in response to CS^–^ presentations in male mice (treatment × tone interaction: *F*[3, 72] = 6.665, *P* = 0.0005) and generalization in both male and female mice (treatment: *F*[1, 24] = 8.585, *P* = 0.0073, and *F*[1, 17] = 5.265, *P* = 0.0348, respectively), without increasing freezing to CS^+^ presentation (Figure 1, G–I, and Supplemental Figure 2, D–F). To further test the relationship between 2-AG levels and fear generalization, we used classical fear conditioning (Figure 1, J–L), and on recall, mice were presented with novel tones (NTs) in addition to CS^+^ presentations. DO34 increased freezing in response to NTs (treatment × tone interaction: *F*[3, 81] = 3.604, *P* = 0.0169 and treatment: *F*[1, 27] = 23.02, *P* < 0.0001) and generalization (treatment: *F*[1, 27]z = 5.684, *P* = 0.0244) in male mice without increasing freezing to CS^+^ presentation. We did not observe any significant differences in female mice (Supplemental Figure 2, G–I). We next expanded our studies to test the hypothesis that 2-AG is required for suppressing contextual fear generalization. Mice were conditioned in context A (CtxA) for 2 days, and on day 3, mice were injected with DO34 and exposed to CtxA or a novel context (NCtx; Figure 1, M–O, and Supplemental Figure 2, J–L). Reducing 2-AG levels via DAGL inhibition increased freezing to NCtx (*F*[1, 33] = 11.03, *P* = 0.0022, and *F*[1, 56] = 41.57, *P* < 0.0001) for male and female mice, respectively) and increased generalization to NCtx (*t*[17] = 2.320, *P* = 0.0330, and *t*[28] = 3.093, *P* = 0.0045). We also observed a significant increase in freezing in response to CtxA in DO34-exposed female mice (Supplemental Figure 2, J–L).

In rodents, the PL has been implicated in reducing fear generalization (11, 15). Given our results with lower basal 2-AG and pharmacological depletion of 2-AG being associated with fear generalization, we hypothesized that 2-AG release in PL is associated with reduced generalization. To test this idea, we used fiber photometry approaches and GRAB-based endocannabinoid sensor (33) in fear-conditioned mice to measure in vivo endocannabinoid release in PL during exposure to NTs (Figure 1, P and Q). As shown in Figure 1, R and S, high fear generalization was associated with lower levels of endocannabinoid release through the duration of the tone and a lower peak magnitude. Our results suggest that 2-AG is required for optimal control over generalization and that 2-AG depletion promotes fear generalization across multiple sensory modalities, with PL being a potentially important locus.

### Prefrontal cortical neuronal activity correlates of fear generalization during 2-AG–deficient states.

**Figure 2:**
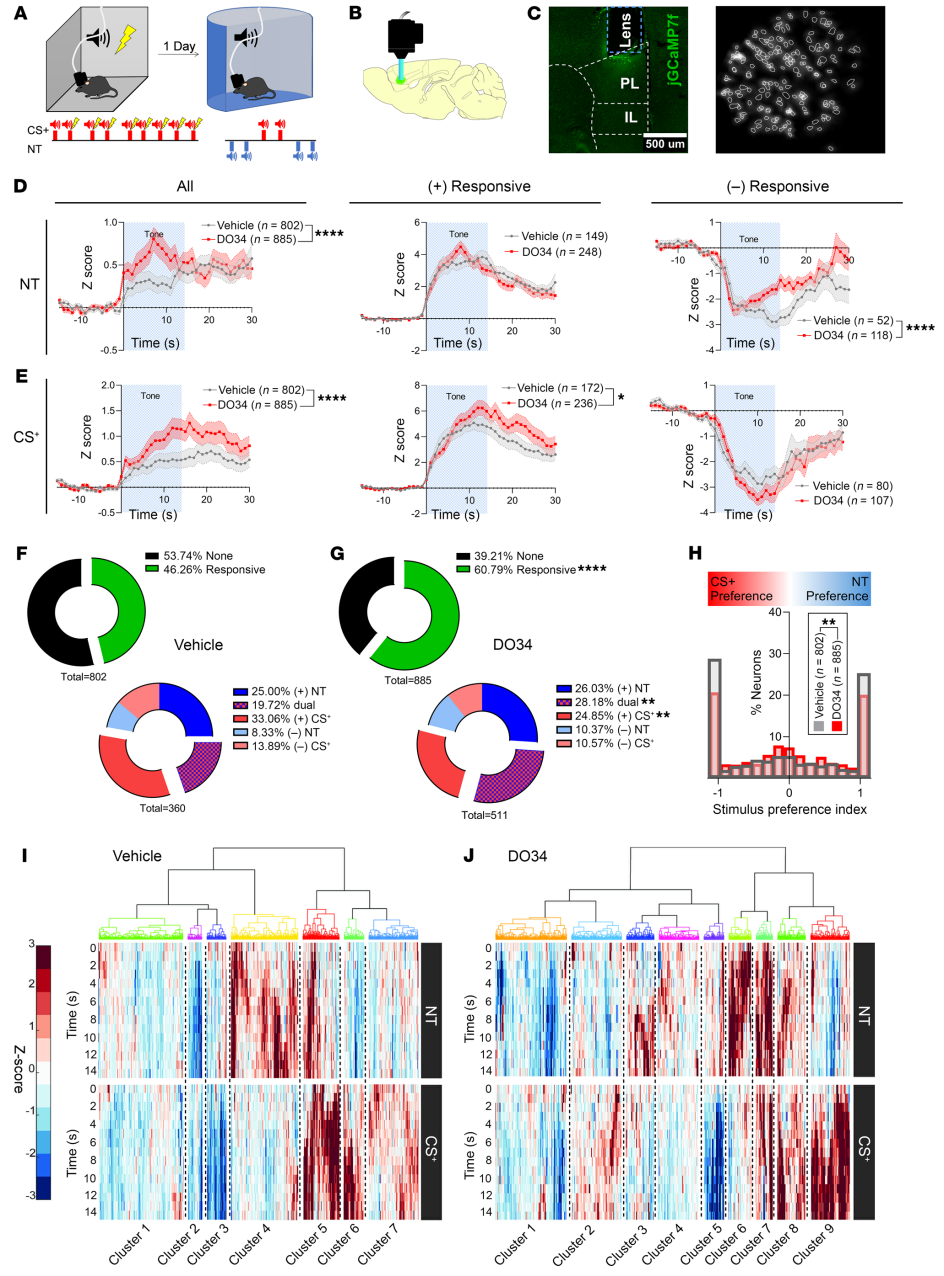
Neuronal tone responses in response to NTs and CS^+^. (**A**) Schematic of experimental timeline and setup. (**B**) Sagittal depiction of GRIN lens and microscope implanted above the region where GCaMP7f was expressed in the PL. (**C**) Left: Example coronal section of the lens track above GCaMP expression in PL. Right: Maximum projection image as seen through the miniaturized microscope, with identified neurons extracted using the CNMFe algorithm surrounded by white lines. (**D**) Left: Average PL neuronal response to NT for all recorded neurons. Middle: NT response for neurons in PL that exceeded +3 *Z*-scores during tone presentation. Right: CS^+^ response for neurons in PL below –3 *Z*-scores during tone presentation. (**E**) Left: Average PL neuronal response to conditioned tone (CS^+^) for all recorded neurons. Middle: CS^+^ response for neurons in PL that exceed +3 *Z*-scores during tone presentation. Right. CS^+^ response for neurons in PL below –3 *Z*-scores during tone presentation. (**F**) Top: Pie charts for vehicle- and (**G**) DO34-exposed mice showing proportion of neurons that significantly responded to tone presentations (±3 *Z*-scores). Bottom: Proportion of neurons that increased activity to NT [(+) NT] or CS^+^ [(+) CS^+^], decreased activity to NT [(–) NT] or CS^+^ [(–) CS^+^], or changed activity to both NT and CS^+^ (dual). (**H**) Histogram of neuronal stimulus preference strength for NT versus CS^+^. Hierarchical tree clustering of neuronal activity to NT and CS^+^ from vehicle- (**I**) and DO34-exposed (**J**) mice. Data are shown as mean ± SEM. Repeated-measures ANOVA followed by Šídák’s multiple-comparison test, Fisher’s exact test, and Kolmogorov-Smirnov test where appropriate. *P < 0.05, **P < 0.01, ***P < 0.001, ****P < 0.0001. *n* = 802 and 885 neurons from *n* = 7 and 8 mice treated with vehicle and DO34, respectively.

The PL has been implicated in both fear expression and generalization, and our results show that 2-AG released in PL may be regulating fear generalization balance (11, 12, 15). However, how PL neurons discriminate between fear-predictive and -nonpredictive stimuli and how this process is affected by low 2-AG levels is not known. To address these questions, we conditioned mice to tones and the following day tested for CS^+^ recall and generalization to NTs after injections of vehicle or DO34 during in vivo single-neuron calcium imaging sessions (Figure 2, A–C). We recorded the activity of 802 PL neurons from 7 mice treated with vehicle and of 885 neurons from 8 mice treated with DO34. DO34 increased net neuronal tone responses relative to pre-tone baseline in response to NT and CS^+^ (main treatment effect: *F*[1, 77,510] = 22.54, *P* < 0.0001, and *F*[1, 77,510] = 76.23, *P* < 0.0001, respectively; Figure 2D). The increased net tone responses could have been mediated by an increase in the magnitude of individual neuronal tone responses. To test this hypothesis, we identified neurons with tone responses exceeding +3 *Z*-scores ([+]responsive, *P* = 0.01, 2-tailed) and with those below –3 *Z*-scores ([–]responsive) in any 2 consecutive 1-second bins during the tone period and examined their respective average responses. This threshold approach has been used by many groups to identify populations of neurons whose activity is modulated by stimuli (12, 34). As shown in Figure 2E, DO34 increased the magnitude of CS^+^ tone responses from (+)responsive neurons (treatment × trial interaction: *F*[45, 18,270] = 1.542, *P* = 0.0114) without changing the magnitude of the responses from (–)responsive neurons. Thus, the increased (+)responsive magnitude appeared to explain the increased net response to CS^+^ tones. Interestingly, DO34 led to a quicker return to baseline from (–)responsive neurons (treatment × trial interaction: *F*[45, 7,560] = 2.050, *P* < 0.0001, and main treatment effect: *F*[1, 168] = 4.945, *P* = 0.0275) without affecting the magnitude of (+)responses.

A larger proportion of tone-responsive neurons could be an important contributor to the higher net neuronal activity to tones seen with DO34. To test this, we formally quantified the proportion of the possible combinations of neuronal tone response types to NT and CS^+^ ([3 possible response patterns]^[2 tone types]^ = 9 response types). We found that DO34 increased the proportion of overall neuronal tone responses (Fisher’s exact test, *P* < 0.00001; Figure 2, F and G). Most importantly, DO34 increased the proportion of dual-tone responsive neurons (Fisher’s exact test, *P* < 0.0052) and decreased the proportion of CS^+^ (+)responses (Fisher’s exact test, *P* < 0.0093) without impacting the proportion of NT-responsive neurons. The profile of dual-responsive neurons was heterogeneous, as shown in Supplemental Figure 3. To further test whether DO34 leads to loss of neuronal NT/CS^+^ selectivity in an unbiased way, we calculated the strength of neuronal preference for either NT or CS^+^ (35). DO34 increased the proportion of neurons that had weak stimulus discrimination between NT or CS^+^ while decreasing the proportion of neurons that had a strong stimulus preference for either NT or CS^+^, consistent with the idea that 2-AG depletion increases neuronal stimulus generalization (Kolmogorov-Smirnov test; *D*[*n*_1_ = 885, *n_2_* = 802] = 0.08250, *P* = 0.0065; Figure 2H). Our results suggest that the observed increase in tone responsivity is explained not simply by increased neuronal excitability but by the increased likelihood of responding specifically to tone presentations, as we actually observed a small reduction in spontaneous calcium events after DO34 treatment (*H*[3] = 47.53, *P* < 0.0001; Supplemental Figure 4). In addition, DO34-treated mice showed a greater proportion of neurons that responded to consecutive tones of any type (Fisher’s exact test: *P* < 0.00001 and *P* < 0.00001 for NT and CS^+^, respectively; Supplemental Figure 5), suggesting that 2-AG depletion increases ensemble stability in response to sensory stimuli presentation. Our results suggest that generalization is associated with an increase in dual-responsive PL neurons that are typically specific to CS^+^, while the proportion of NT active neurons remains stable.

To gain additional insight into the diversity of neuronal responses to distinct tone types, we conducted agglomerative hierarchical clustering of neuronal tone responses. We observed large heterogeneity of responses with clusters of neurons that did not respond to tones, neurons that exclusively responded to one tone type, and neurons with diverse types of dual responses to tones (Figure 2, I and J). Notably, we found clusters of CS^+^-specific neurons and low-grade NT-modulated neurons in the vehicle group and clusters of NT-specific neurons and NT/CS^+^ dual-activation neurons in the DO34 group (Supplemental Figures 6 and 7). We also found a drastic reduction in cluster size for clusters with CS^+^ specific neurons in the DO34 relative to the vehicle group.

Upon analysis of neurons longitudinally registered between conditioning and recall, we found that neuronal tone or shock responsiveness during conditioning did not predict tone responsiveness on recall, as hierarchical agglomerative clustering only revealed a small cluster of tone/shock-responsive neurons that also responded to tones during the recall (Supplemental Figure 8). Moreover, only approximately 4% of neurons that responded to CS^+^ during the late conditioning phase responded to NTs or CS^+^ during recall. These data suggest a high degree of ensemble drift across conditioning and recall with respect to tone-responsive neurons in PL. This finding contrasts with evidence that amygdala ensembles are more stable across days following fear conditioning (36).

**Figure 3:**
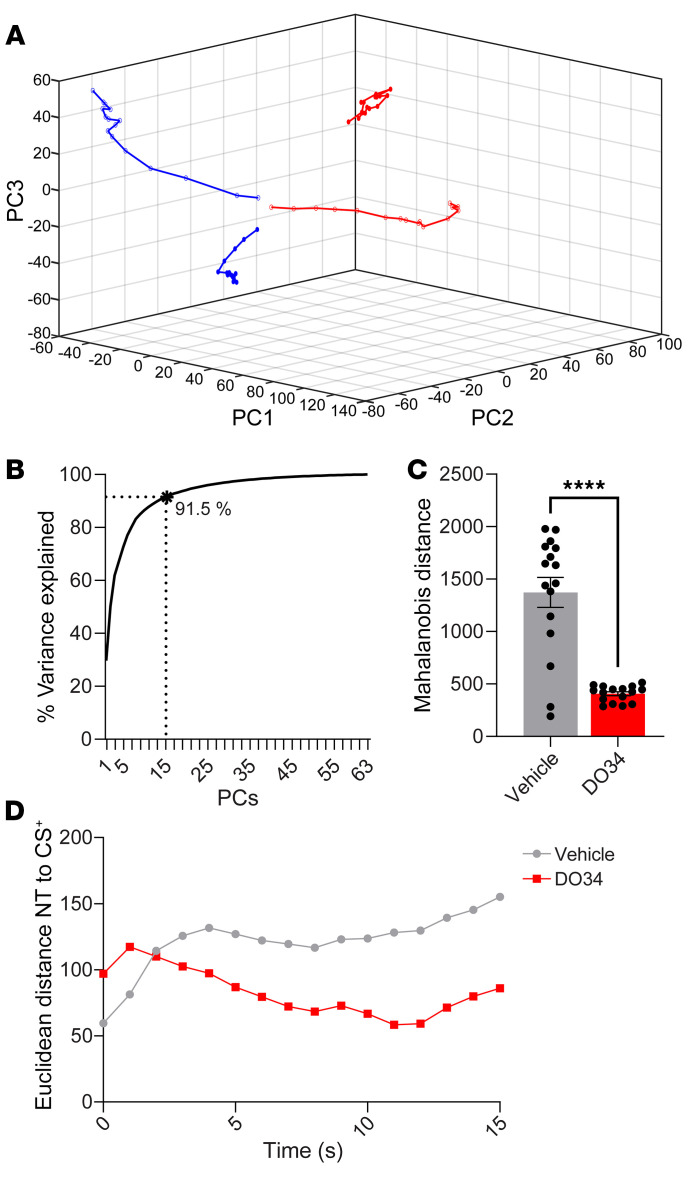
Reduced 2-AG is associated with increasing similarity of population metrics. (**A**) Trajectory of neuronal population activity in 3D PC space during presentation of the CS^+^ (open dots) and NT (filled dots) for both DO34- (red) and vehicle-treated (blue) mice. (**B**) Fifteen PCs explain 91.5% of the variance in the neuronal data set. (**C**) Mahalanobis distance between NT and CS^+^ trajectories in PC space for vehicle- and DO34-exposed mice. (**D**) Instantaneous Euclidean distance between NT and CS^+^ trajectories across tone duration. Student’s *t* test. ****P < 0.0001.

Our data above indicating an increase in dual-responsive neurons after 2-AG depletion suggests that DO34 could have increased generalization by increasing the similarity of the population activity associated with NT and CS^+^ presentations. To address this hypothesis, we used a population vector approach and principal component analysis (PCA) for dimensionality reduction of neuronal activity during tone presentations. Figure 3A depicts the trajectory of population activity in 3D principal component (PC) space during presentations of the CS^+^ and NT for both DO34- and vehicle-treated mice. We used 15 PCs, which accounted for 91.5% of the variance in our data set, for our analysis (Figure 3B). We then formally quantified the distance between NT and CS^+^ in PC space. As shown in Figure 3C, DO34 decreased the Mahalanobis distance between NT and CS^+^ activity in PC space relative to vehicle treatment (*t*[30] = *t* = 6.705, *P* < 0.0001). Moreover, despite starting at a higher level, the Euclidean distance between NT and CS^+^ activity trajectories decreased across tone presentations in DO34-treated mice, while the activity distance for the vehicle group continued to rise (Figure 3D). These results suggest that DO34 increased the similarity in population activity dynamics between NT and CS^+^, which could represent an important neural correlate of fear generalization.

### Prefrontal cortex–amygdala connectivity is inversely correlated with fear generalization.

**Figure 4:**
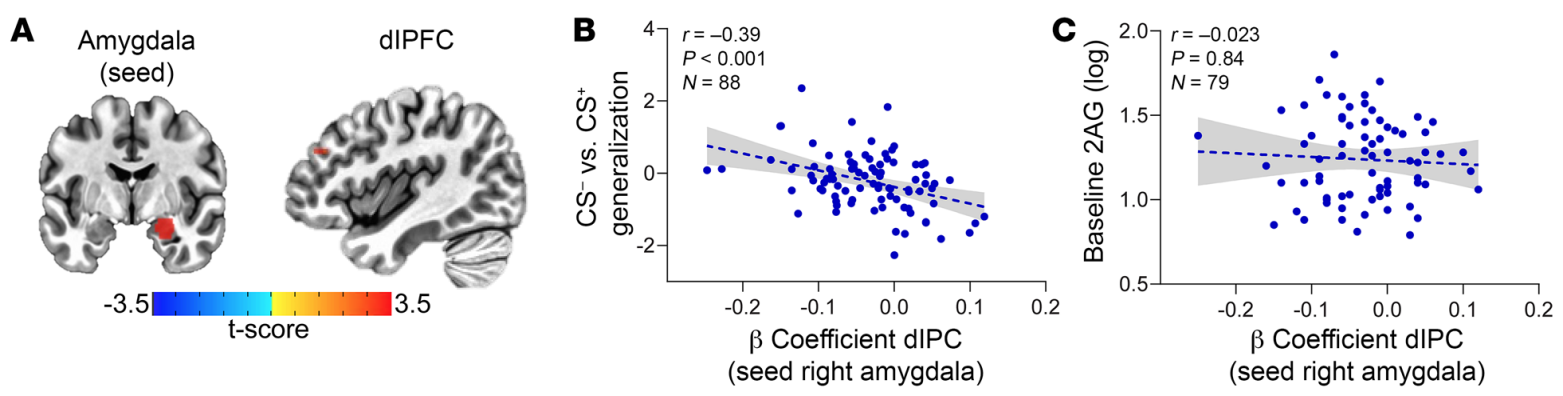
dlPFC-amygdala resting-state connectivity is inversely correlated to fear generalization. (**A**) Depiction of increased resting state between the right amygdala and dlPFC. (**B**) Generalization between CS^–^ and CS^+^ as a function of β coefficient for dlPFC. (**C**) Baseline 2-AG levels as a function of β coefficient for dlPFC.

Our results implicate the prefrontal cortex (PFC) in fear generalization/discrimination, and amygdala-PFC connectivity has been implicated in fear expression and reduced generalization (11, 15, 37, 38). To explore whether fear generalization and 2-AG levels were associated with basal amygdala-PFC connectivity, we obtained and analyzed brain resting-state data. Therefore, the amygdala was used as a seed to identify PFC regions that may be functionally connected to the amygdala in an unbiased way (Figure 4A). β Coefficients for the right/left amygdala were assessed for covariance with peripheral 2-AG levels and fear generalization. As shown in Figure 4B, CS generalization explained decreased connectivity between the right amygdala and dorsolateral PFC (dlPFC). Specifically, we identified a positive cluster in dlPFC (area 46, MNI: –44, 37, 28, 5 voxels), with CS generalization as a covariate (Figure 4B). We did not observe any significant association between 2-AG levels and amygdala-dlPFC connectivity (Figure 4C) or amygdala–anterior cingulate cortex (amygdala-ACC) connectivity. We also did not observe a significant association between fear generalization and amygdala-ACC connectivity.

## Discussion

Using distinct but complementary approaches we show, in experiments with both mice and human participants, that 2-AG regulates the balance between fear discrimination and generalization. Plasma levels of 2-AG were inversely correlated with generalization in humans, and pharmacological 2-AG depletion increased fear generalization in mice under diverse behavioral paradigms. Moreover, this increased generalization was associated with impaired sensory discrimination within PL neuronal ensembles responsive to sensory cues and decreased connectivity between the right amygdala and dlPFC in humans.

We show that plasma levels of 2-AG predicted discriminatory performance without affecting CS^+^ responses. There are three potential explanations for this: (i) spillover of tonic neurotransmitter release, which could have predictive value over subsequent task performance; (ii) permeation of peripherally produced 2-AG across the blood-brain barrier and into neuronal synapses, which in turn has a modulatory effect over behavior; and (iii) conserved (central-peripheral) within-subject enzymatic balance between production and degradation of 2-AG that is reflected by plasma levels and varies depending on individual environmental, developmental, genetic, and epigenetic factors. Of these, the first possibility is less likely, as mice with DAGLα knockout have normal levels of plasma 2-AG despite having very low brain 2-AG levels; thus, brain levels seem not to have an effect over plasma levels (17). The second possibility is also less likely, as synaptic 2-AG signaling is tightly regulated by local synthesis and degradation on the time scale of seconds, which is unlikely to be affected by relatively slow changes in circulating 2-AG. Ultimately, the mechanistic link between peripheral and brain levels of 2-AG and other endocannabinoids still needs to be established.

We have previously explored endocannabinoid function in relation to fear learning in healthy control subjects (39, 40) and did not find a relationship between 2-AG levels and fear learning. Subjects participating in this study had a differential history of trauma and substance use. Indeed, this heterogeneity may be required for obtaining larger variability and observing a relationship between 2-AG and fear generalization, as this was not observed during the analysis of participant subsets. This may provide further support for the third hypothesis noted above. We suggest that including differential history of SUD and CM within our study sample created a more accurate representation of the general population than that obtained when only including participants with no history of childhood trauma exposure or current psychiatric diagnoses. In particular, 15%–43% of adults are exposed to trauma during childhood or adolescence (National Center for PTSD), and one-third of adults in the United States will meet the criteria for an alcohol use disorder in their lifetime (41). Thus, the results we provide may be more representative of real-world adults and provide insights into endocannabinoid-trauma interactions that would likely be difficult to predict in a true control sample.

Previous studies examining peripheral 2-AG levels and PTSD symptoms have been mixed (21, 22, 25–28, 30); some have shown an association between lower levels of 2-AG and PTSD diagnosis after traumatic stress exposure (21, 28). In contrast, higher 2-AG levels immediately after trauma exposure were associated with subsequent PTSD diagnosis and positively correlated with symptom severity in racial minority individuals (42). Future studies aimed at more clearly defining the relationship between peripheral and central 2-AG levels; between peripheral 2-AG levels and fear learning, both dynamically and temporally; and between 2-AG levels and aspects of trauma and PTSD will be critical for resolving these apparent discrepancies in the literature.

2-AG production is regulated by activity and executed on demand to suppress presynaptic neurotransmitter release via activation of cannabinoid receptor 1 (CB1R). DAGLα is induced to produce 2-AG following neuronal activity-related increases of intracellular calcium as well as activation of metabotropic glutamate receptors or muscarinic cholinergic receptors, among others. In turn, 2-AG is degraded by monoacylglycerol lipase (MAGL). It is possible that our findings implicating reduced DAGL activity and 2-AG levels in fear generalization may extend to other vulnerable points in the 2-AG production/CB1R activation/2-AG degradation axis. Potentially, any reduction in 2-AG/CB1R activity may lead to increased fear generalization. For instance, single nucleotide polymorphisms affecting CB1R have been implicated in fear extinction failure and higher fear generalization in individuals with PTSD (43) and could be a risk factor for developing PTSD (44, 45). It is possible that increased MAGL activity from unidentified genetic variants or epigenetic changes may also lead to decreased 2-AG and reduced CB1R activation and subsequent fear generalization.

The dlPFC has been implicated in emotional regulation, acquisition and expression of fear, and reduced generalization/improved discrimination (46, 47). It is important to note that the dlPFC lacks a direct rodent homolog; however, dlPFC connects directly with regions that are involved in emotional regulation such as the dorsal ACC (dACC) and ventromedial PFC, which have known rodent homologs, and also functionally connects with the amygdala (48, 49). Nevertheless, our results further implicate the PFC in discrimination between safety and danger, as PFC-amygdala connectivity was predictive of discrimination between safe and aversive stimuli. Intriguingly, we found an association between dlPFC-amygdala, but not ACC-amygdala, connectivity and fear generalization. In part, this finding could be related to the fact that our data were obtained during the resting state, whereas studies implicating ACC in fear expression and functional homology to the rodent PL characterize dACC activity in response to cue presentations (50–52). The dlPFC does not directly connect with the amygdala, and it has been proposed that the dlPFC exerts cognitive control over amygdala activity through the ventromedial PFC (53–55). The dlPFC also targets the dACC (56, 57) and in turn, the dACC targets the amygdala (58, 59); we suggest that the dlPFC/dACC/amygdala circuit could provide an important route to exert control over amygdala neutral stimuli responses and reduce fear generalization. Our findings implicating dlPFC/amygdala/resting-state connectivity and decreased generalization may suggest that resting-state connectivity could be an intrinsic indicator of potential entrainment strength of dlPFC over the amygdala to suppress generalized threat responses. These data also suggest that resting-state functional connectivity between PFC and amygdala could be useful as a biomarker with predictive value for fear generalization or even development of PTSD, though confirmatory studies are warranted. One potential limitation of our study is that the imaging session was conducted on a separate day from the fear conditioning; thus, it is unclear whether resting connectivity would be different from that obtained prior to or during fear conditioning.

In rodents, the ventromedial PFC has been implicated in reducing fear generalization (11). Specifically, the infralimbic PFC regulates safety learning and reduction of conditioned fear and generalization (8, 60–64). The prelimbic cortex mediates conditioned fear expression, safety learning, and reduction of fear generalization (8, 12–15, 65–67). Our results show that PL contains information needed for reducing generalization, as loss of specificity in PL CS^+^ neuronal ensembles was associated with fear generalization. This idea is supported by another study indicating that the overlap (and associated behavioral generalization) between the CS^+^- and NT-activated PL neuronal subpopulations increases as the similarity in frequency between the two stimuli increases (68). Thus, neuronal ensembles within PL simultaneously representing both auditory sensory stimuli are embedded in PL, and fear generalization may be a melding of both populations and concurrently increased similarity between NT and CS^+^ population dynamics.

There are several intriguing and testable possibilities for how the expansion of neuronal ensembles in PL representing fear memory generalization is constrained by 2-AG: (i) Local interneuron disinhibitory networks filter ensemble size upon stimulus presentation, and in the absence of 2-AG PL neurons that respond to CS^+^ are more likely to respond to other stimuli; thus, the generalizing ensemble expands. (ii) In the absence of 2-AG, reduced stimulus filtering leads to expansion of neuronal ensembles that generalize within PL afferent structures such as the amygdala or ventral hippocampus, which in turn feed into PL neurons to guide expanded generalization ensembles. A previous study of amygdala neurons has demonstrated the expansion of ensembles encoding generalization in the amygdala with correlated increases in fear generalization (6). In the absence of negative feedback mediated by 2-AG, PL afferent neurons can have a stronger excitatory drive over PL neurons, and thus PL neurons are more likely to activate when presented with generalizable stimuli, leading to expansion of the generalization ensemble. A recent study using contextual fear generalization found that two nonoverlapping subsets of genetically defined neurons in the dentate gyrus that predominantly receive inputs from medial entorhinal cortex and dentate gyrus cholecystokinin^+^ interneurons mediate contextual fear generalization and discrimination, respectively (69). Our data suggest that in PL, rather than nonoverlapping populations mediating the balance between discrimination and generalization, the balance is determined by the likelihood of CS^+^ specific neurons being recruited to respond to the generalized stimuli. While our study does not establish causality by directly targeting PL activity or overlapping NT/CS^+^ ensembles, recent studies demonstrate that disrupting activity in PL and its projection to basolateral amygdala and periaqueductal gray increases fear generalization (14, 15), further supporting the idea that PL may have a dynamic role in fine-tuning the balance between generalization and discrimination.

Previous studies in rodents have shown sex differences in fear generalization and expression of other conditioned responses (70–72). Our study did not find any sex differences in generalization or 2-AG measures in human participants. In part, this difference could be attributed to the conditioning protocol used, interspecies differences, or modest sample size, which may have precluded adequate formal testing for such differences. Despite the lack of sex differences in humans, one limitation of our study is that we only examined the neuronal activity of male mice, as these exhibited more consistent pharmacological effects in fear generalization. Female mice exhibited fear generalization when 2-AG was depleted under some of the fear-conditioning protocols used; however, higher baseline fear generalization may have occluded observation of generalization to NTs following pharmacological depletion of 2-AG as was seen in males. Higher levels of fear generalization in female mice have been observed in previous studies (71, 73).

The diagnosis of PTSD requires that symptoms persist for weeks following the traumatic experience, which suggests that differences in systems consolidation processes of the original trauma-associated memories may be important to its pathogenesis; thus, the lack of longitudinal memory testing in our study was a limitation. However, differences in early fear memory acquisition and consolidation and resulting memory strength may also be important for the pathogenesis of PTSD, and understanding these may ultimately allow for early identification of at-risk subjects and intervention. Indeed, genetic variants associated with increased emotional memory strength have been associated with PTSD symptoms (74, 75). Our study provides insight into how fear generalization is represented in the PFC and how 2-AG signaling contributes to this process. These data provide cross-species evidence that 2-AG plays a critical role in opposing fear generalization and support the notion that 2-AG–deficient states may contribute to the development of trauma-related disorders including PTSD. Intriguingly, our results suggest that plasma 2-AG levels and dlPFC-amygdala connectivity may serve as independent biomarkers related to fear generalization. Future studies may focus on assessing whether these parameters, at least at the lowest or highest extremes, are predictive of resilience or susceptibility to trauma.

## Methods

### Sex as a biological variable.

Human experiments included both male and female subjects. Rodent studies were segregated by sex. Results of experiments with female mice are included in the supplemental figures. Calcium imaging experiments only included male mice, as female mice overgeneralized conditioned fear to NTs, which occluded observation of drug effects.

### Study overview.

The human study consisted of one screening visit and two laboratory sessions. During screening, participants were evaluated for eligibility and completed questionnaire measures, including the Difficulties in Emotion Regulation Scale (76) (see Figure 1A for study schematic). In the first laboratory session, blood samples and psychophysiological recordings were collected, and participants completed behavioral tasks, including fear conditioning and extinction. The second session included an MRI session, with one anatomical, one post-recall resting-state, and three task-based scans (for other tasks not included here, see ref. 77).

### Human participants.

Participants were recruited between March 2017 and July 2020 at Linköping University. A total of 101 participants were included in the study, divided into 4 groups across the dimensions of histories of CM and SUDs (described further in Supplemental Methods and refs. 77, 78). However, there were no group differences across the measures described below, so all analyses were collapsed across groups for a total of *n* = 101 (see Table 1 for demographic information). Of note, individuals with histories of CM were recruited via prior contact with a specialized trauma unit within Child and Adolescent Psychiatry at the regional hospital in Linköping, which was created in 1999. As a result, the upper age limit of patients was dependent on being a former patient of the trauma unit since its inception, while the lower age limit was 18.

Before laboratory sessions, participants completed breath and urine screens for alcohol and drug use. During the screening session, participants underwent a psychiatric clinical assessment by a trained research nurse or study physician. After determination of eligibility, participants received research information, provided written informed consent, and completed self-report questionnaires. Detailed descriptions are provided in Supplemental Methods.

### Human fear conditioning.

Upon arrival at the laboratory, participants were fitted with an intravenous catheter for blood sample collection for subsequent analysis of endocannabinoid levels and prepared for psychophysiological recordings via the application of facial electromyography (EMG) recording electrodes on the orbicularis muscle to assess the eye blink component of the startle response. EMG data collection and fear conditioning took place as previously described (39, 40) and are detailed in Supplemental Methods. Briefly, the fear-conditioning task consisted of habituation, acquisition, and recall, with the first 4 trials of extinction serving as the CS generalization test (79). In the acquisition step, a specific conditioned stimulus (lamp color, CS^+^) predicted an unpleasant sound (US; nails across the chalkboard; ref. 80), while another CS (different lamp color, CS^–^) was not followed by any stimulus. After a 10-minute break, recall took place in a different context (different picture on the computer), with the same CS^+^ and CS^–^ but no US. A startle probe was used to elicit blinking during each CS and intertrial interval (ITI), measured as the peak-to-peak EMG value. Startle responses to the CS^+^ were standardized to the mean startle response during ITI/rest trials to account for individual differences in startle magnitude (39, 40, 81, 82). Due to technical issues (i.e., inadvertent partial removal of the EMG sensor during task completion), *n* = 3 individuals did not have complete fear-conditioning data and thus were not included in the analysis.

### Endocannabinoid analysis.

Baseline endocannabinoid levels were calculated as the average of two time points. AEA and 2-AG were extracted and analyzed using liquid chromatography–tandem mass spectrometry (LC-MS/MS), as previously published (39, 83) (Supplemental Methods). Endocannabinoid values were log-transformed due to the non-normality of the distribution; these transformed values were used in all subsequent analyses. Baseline differences in endocannabinoids were added as covariates in the analysis of brain, behavioral, and self-report measures. Of the *n* = 101 participants, technical issues prevented blood from being drawn in *n* = 13. An additional *n* = 6 had 2-AG levels below the lower limit of detection. Thus, analyses of endocannabinoid levels included *n* = 82 participants. A total of *n* = 3 participants were removed from the fear-conditioning analysis (of them, *n* = 1 did not have endocannabinoid data). Thus, *n* = 80 participants had complete fear-conditioning and endocannabinoid data.

### MRI.

MRI acquisition and analysis steps are presented in detail in Supplemental Methods. Briefly, anatomical and functional blood oxygen level–dependent (BOLD) data were collected on a Siemens MAGNETOM Prisma 3T MRI scanner (Siemens Healthcare AB) equipped with a 64-channel head coil. Echo planar images were de-spiked, slice-time corrected, smoothed using a 4 mm kernel, and motion-corrected. Preprocessing and statistical analysis were performed with AFNI software v18.3.16 (33).

For each participant (*n* = 88), the amygdala time course was entered as a predictor in a regression analysis on preprocessed data, using 3dDeconvolve. Resulting 3D volumes with β coefficients for right/left amygdala were assessed for covariance with peripheral measures of endocannabinoid function and behavioral measures of fear conditioning using the AFNI function 3dttest++ and with 2AG (*n* = 73) and CS generalization (CS^–^ vs. CS^+^) at recall (*n* = 88) as covariates in two separate analyses. The target region included dlPFC, known to be involved in fear conditioning in humans (46, 47); and the ACC, including area 24 in its dorsal portion, considered to be the human homolog of the prelimbic cortex in rodents. The two regions were defined using the Matthew Glasser and Brainnetome atlases contained within Analysis of Functional NeuroImages (AFNI) software (https://afni.nimh.nih.gov), respectively (Supplemental Figure 9). Activation maps were thresholded at per-voxel *P* < 0.002, cluster corrected at α = 0.05 (36). To visualize the significant correlations, β coefficients from the significant cluster and the corresponding covariate are presented in two scatter plots.

### Rodents.

A total of 135 male and 88 female C57BL/6J mice older than 8 weeks and obtained from The Jackson Laboratory or bred in our animal facility were used in our experiments. Mice were housed in a temperature- and humidity-controlled housing facility under a 12-hour light/12-hour dark cycle with ad libitum access to food. Mice were group housed, except for mice implanted with gradient-index (GRIN) lenses, which were single-housed. Statistical analysis and graphing were performed using GraphPad Prism 9, and the analysis performed is described in the figure legends as appropriate.

### Rodent surgeries.

Briefly, mice were anesthetized with isoflurane and mounted into a stereotax. Skull hairs were trimmed, and overlying skin was cleaned with 70% isopropyl alcohol and iodine. A midline incision was performed, and a small hole was drilled above the PL to allow for injection of AAV encoding GCaMP7f under control of the synapsin promoter (AAVrg-syn-jGCaMP7f-WPRE; Addgene). Following injection, a GRIN lens was lowered into the PL and was cemented to the skull. For fiber photometry experiments, mice were injected with AAV encoding the endocannabinoid sensor GRABeCB2.0 under control of the synapsin promoter (AAV9-hSyn-GRABeCB2.0; BrainVTA), and 400 μM fiberoptic cannula (Doric Lenses) was implanted above the PL. Mice were allowed to recover for at least 2 weeks. More detailed information is provided in Supplemental Methods and Patel et al. (84).

### Rodent behavior and recordings.

Mice were exposed to fear-conditioning protocols as depicted in Figure 1 and described in detail in Supplemental Methods. For auditory fear conditioning, foot shocks were 0.4 mA in intensity and 1 second in duration. 2 kHz and white noise tones at 75 dB were counterbalanced across experiments to control for stimulus effects. For contextual fear conditioning, mice were exposed to 2 sessions in the conditioning context (CtxA), 270 seconds in duration, 1 per day, with 2 0.4 mA foot shocks, 2 seconds in duration. Mice were injected with DO34 before the recall phase of conditioning experiments. The recall phase was conducted 2 hours following DO34 injections and was carried out in a modified conditioning chamber (CtxB). For contextual fear recall, mice were exposed to the modified context as the NCtx or to CtxA.

Fiber photometry recordings were conducted during fear conditioning as described above and depicted in Figure 1, P and Q. The patch cord was attached before behavioral sessions. A 465nm LED (Lx465; Tucker Davis Technologies [TDT]) was used to excite the endocannabinoid sensor and detect GFP-dependent changes in fluorescence; a 415 nm LED (Lx415; TDT) was used to excite the endocannabinoid sensor at its isosbestic point. More detailed information can be found in Supplemental Methods and Kondev et al. (85).

Calcium imaging experiments were conducted during fear conditioning as described above and depicted in Figure 3A. The miniaturized microscope (Inscopix) was attached before behavioral sessions. On recall day, home cage recordings were conducted for 15 minutes before and 2 hours after DO34 injections. Following home cage recording sessions, mice were transferred to CtxB and exposed to the recall protocol. Data were acquired at a sampling rate of 10 Hz. All the fear-conditioning experiments were controlled using FreezeFrame (Actimetrics). For calcium activity and fiber photometry recordings, the calcium imaging data acquisition or photometry data acquisition system was interfaced with FreezeFrame using custom-made RJ12 connector to BNC connector cables to deliver transistor-transistor logic (TTL) pulses synchronizing the start of the experiment with the start of data acquisition and tone deliveries.

### Histology.

Following completion of calcium imaging or endocannabinoid sensor experiments, mice were anesthetized using isoflurane and transcardially perfused with ice-cold PBS, followed by 4% paraformaldehyde (PFA) solution in PBS. Brains were extracted and kept overnight in 4% PFA and transferred to 30% sucrose for 1 day for cryoprotection. Coronal sections (40 μm) were obtained using a CM3050 S cryostat (Leica Microsystem), mounted on glass slides, and cover slipped. Images were taken using an Axio Imager M2 epifluorescence microscope coupled to a fluorescent lamp. Expression of GCaMP7f or endocannabinoid sensor and GRIN lens or optic fibers were confirmed to be localized to PL.

### Rodent data analysis.

Upon completion of experiments, freezing data were extracted from FreezeFrame. The time spent freezing in response to tones or context was expressed as a percentage of the interval. Freezing to tones is shown in blocks of 2 tones. For fiber photometry data, ΔF/F signals calculated from the active sensor and isosbestic signals from 7 mice were downsampled to 10 Hz using custom MATLAB codes. The traces were then aligned to tones and *Z*-scored using the 2-second pre-tone baseline for normalization. *Z*-scored signals corresponding to tones that elicited more than 75% or less than 25% freezing and their peak value were pooled together.

Calcium imaging data were analyzed as previously described (84, 86). Briefly, data were preprocessed, bandpass filtered, and motion corrected using Inscopix Data Processing software V1.3. Individual neuron calcium traces were extracted using constrained non-negative matrix factorization for endoscopy algorithm (CNMFe) (87), and false positives were excluded from further analysis. The individual traces were aligned to tone onset and binned into 1-second intervals. The binned traces were then averaged into blocks of 2 and normalized using *Z*-transform using the 15-second pre-tone period as a normalization baseline. Traces exceeding a *Z*-score value of 3 or –3 (*P* = 0.01, 2-tailed) for any 2 consecutive bins following tone onset during the tone period were considered (+)responsive or (–)responsive, respectively.

Neuronal stimulus preference strength for either NT or CS^+^ was defined as |*Z_NT_* – *Z_CS+_*|/(|*Z_NT_*| + |*Z_CS+_*|), where *Z_NT_* and *Z_CS+_* are the average *Z*-scores during the NT and CS^+^ presentation periods for the corresponding neuron. Average *Z*-scores were obtained by applying the *Z*-transform to an average of 2 stimulus presentations of each type, using the whole session activity as the normalization baseline and then averaging the *Z*-score for the 15 seconds of stimulus duration. If |*Z_NT_*| was greater than |*Z_CS+_*|, the index was defined as a positive value, whereas if |*Z_NT_*| was less than |*Z_CS+_*|, the index was defined as a negative value. This preference index was calculated for each neuron within the treatment group.

Agglomerative hierarchical clustering was conducted using the available MATLAB (MathWorks) function clustergram. *Z*-scored traces for NT and CS^+^ were rescaled using the available MATLAB function rescale, and –3 and 3 were used as parameters, with –5 and 5 as input minimum and maximum. This facilitated clustering by removing bias from traces with relatively large *Z*-score changes while preserving the pertinent activity pattern. The clustergram function was then applied to the rescaled traces. Linkage was then calculated using Ward’s method, with Euclidean distance for metric. 5% of the maximum linkage was used as a threshold to form distinct clusters within the resulting dendrogram. The resulting clusters were then remapped to the unscaled *Z*-score traces to accurately represent the activity patterns and magnitude for each cluster.

PCA was conducted using available MATLAB functions. *Z*-scored traces for NT and CS^+^ periods were concatenated for vehicle and DO34 groups. Concatenated traces for the DO34 group were randomly selected to create a matrix that had the same number of traces as the one for the vehicle group. The resulting vehicle and DO34 matrices were then concatenated, and PCA was applied across the dimension of the activity traces. For visualization, the first 3 PCA dimensions for each group and stimulus type were plotted. To assess the distance between NT and CS^+^ in PC space, the 15D Mahalanobis distance between the NT and CS^+^ traces was calculated for vehicle and DO34 groups. To calculate the distance between NT and CS^+^ across the tone duration, we calculated the instantaneous Euclidean distance between the NT and CS^+^ for each 1-second bin for the first 15 components in PC space.

### Statistics.

Data from human experiments were analyzed using Statistical Package for the Social Sciences (SPSS) software version 28.0.1.0, and graphs were created in GraphPad Prism 9. For rodent experiments, statistical analysis and graphing were performed using Prism 9, and the analysis was performed is described in the figure legends. In general, linear regression, Kolmogorov-Smirnov test, 2-tailed Student’s *t* test, repeated-measures ANOVA followed by Šídák’s multiple-comparison test, or 2-way ANOVA followed by Šídák’s multiple-comparison test were performed where appropriate. *P* values less than 0.05 were considered statistically significant.

### Study approval.

The study with human participants was approved by the Regional Ethics Review Board in Linköping, Sweden (Dnr 2015/256-31, and 2017/41-32). Participants provided written informed consent prior to participation in the study. All rodent experiments were approved by the Vanderbilt University and Northwestern University Institutional Animal Care and Use Committees and were conducted in accordance with the *Guide for the Care and Use of Laboratory Animals* (National Academies Press, 2011).

### Data availability.

Data for all data points in graphs are reported in the Supporting Data Values file. Custom codes used for analysis are available from GitHub (https://github.com/luisrosasvidal/Rosas-Vidal2025; commit ID d06965f).

## Author contributions

LERV, SN, LMM, IP, MH, and SP conceived the study, designed experiments, and cowrote the manuscript. LERV, SN, LMM, IP, RM, MA, LRM, SDZ, HE, and PJ performed experiments and acquired and analyzed data.

## Supplementary Material

Supplemental data

Supporting data values

## Figures and Tables

**Table 1:**
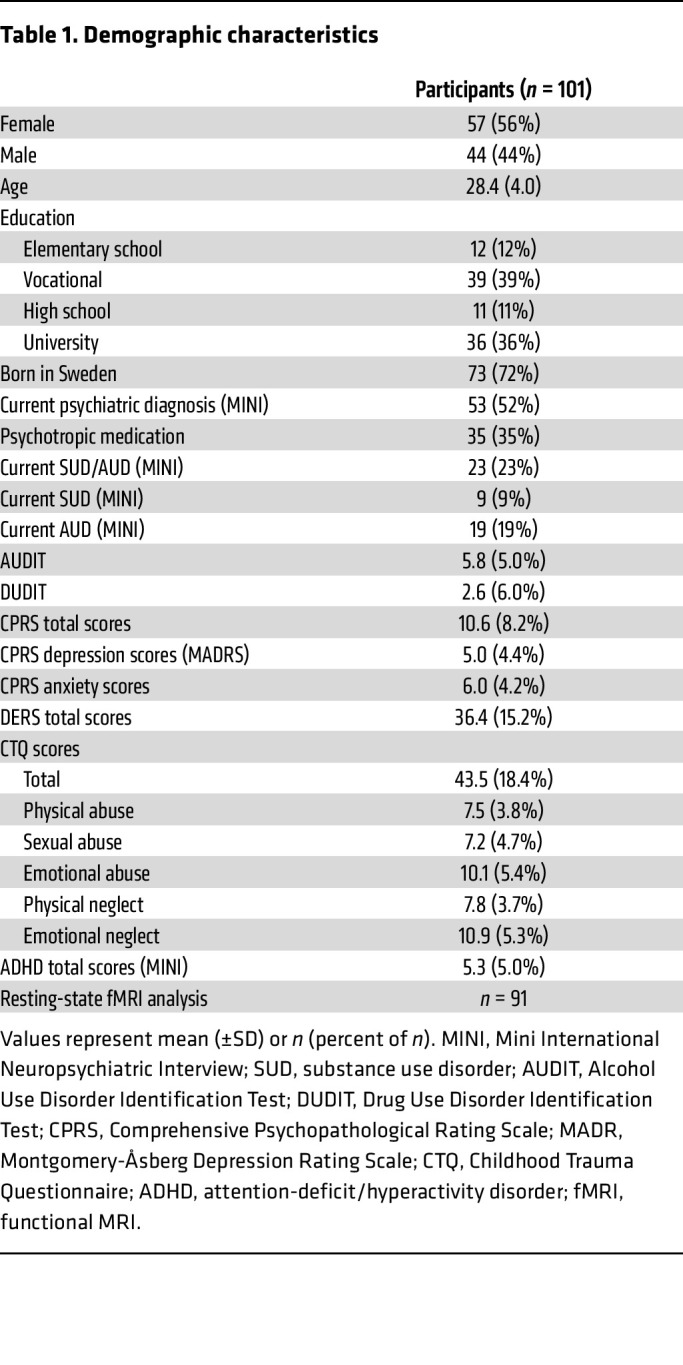
Demographic characteristics

## Version 1 03/27/2025

In-Press Preview

## Version 2 06/02/2025

Electronic publication
